# Childhood socio-economic circumstances and dementia: prospective register-based cohort study of adulthood socio-economic and cardiovascular health mediators

**DOI:** 10.1093/ije/dyac205

**Published:** 2022-11-07

**Authors:** Kaarina Korhonen, Taina Leinonen, Lasse Tarkiainen, Elina Einiö, Pekka Martikainen

**Affiliations:** Population Research Unit, Faculty of Social Sciences, University of Helsinki, Helsinki, Finland; Finnish Institute of Occupational Health, Helsinki, Finland; Population Research Unit, Faculty of Social Sciences, University of Helsinki, Helsinki, Finland; Helsinki Institute of Urban and Regional Studies (URBARIA), University of Helsinki, Helsinki, Finland; Population Research Unit, Faculty of Social Sciences, University of Helsinki, Helsinki, Finland; Population Research Unit, Faculty of Social Sciences, University of Helsinki, Helsinki, Finland; Max Planck Institute for Demographic Research, Rostock, Germany; Department of Public Health Sciences, Stockholm University, Stockholm, Sweden

**Keywords:** Alzheimer’s disease, cardiovascular health, life course, mediation analysis, socio-economic status

## Abstract

**Background:**

This study analysed the association between childhood socio-economic circumstances and the risk of dementia, and investigated the mediating role of potentially modifiable risk factors including adulthood socio-economic position and cardiovascular health.

**Methods:**

We used a 10% sample of the 1950 Finnish population census linked with subsequent population and health registers (*n* = 95 381). Information of socio-economic characteristics, family structure and housing conditions at the age of 0–15 years was obtained from the 1950 census. We identified cohort members who developed dementia in 2000–2018 using national hospital, medication and death registers. Discrete time survival analysis using logistic regression and mediation analysis applying the Karlson–Holm–Breen (KHB) method were employed.

**Results:**

An excess risk of dementia was observed for household crowding [odds ratio (OR) = 1.10; 95% CI 1.02–1.18 for 3 to <4 persons per heated room; OR = 1.19; 95% CI 1.11–1.27 for ≥4 persons], single-father family (OR = 1.27; 95% CI 1.07–1.51) and eastern and northern region of residence (OR = 1.19; 95% CI 1.10–1.28). The effects of single-father family and region of residence were mostly direct with adulthood characteristics mediating 14% and 29% of the total effect, respectively. The largest indirect effect was observed for household crowding mediated through adulthood socio-economic position (47–65%).

**Conclusions:**

The study shows that childhood socio-economic circumstances are associated with dementia, and that the underlying mechanisms only partly relate to adulthood socio-economic position and cardiovascular health. Socio-economic and health interventions targeted at families with children may carry long-term benefits by contributing to a lower dementia risk in later life.

Key Messages

- Prior evidence of the association between childhood socio-economic circumstances and dementia is scarce and mixed, and mostly relies on retrospective self and proxy reports of childhood circumstances.
- This study used prospectively collected census and population register data, and identified an increased risk of dementia among people having lived in crowded households, with a single father and in the mostly rural eastern and northern regions of Finland.
- Formal mediation analysis showed that adulthood socio-economic position and cardiovascular health were important mechanisms in linking household crowding and dementia, but less so in linking family type and region of residence with dementia.
- Results suggest that the life-course social determinants are similar for early-onset dementia as for dementia overall.
- Preventive interventions should be targeted at families with children to reduce the excess risk of dementia associated with disadvantaged childhood circumstances.

## Background

A rich body of literature shows that childhood socio-economic circumstances affect cardiovascular morbidity and mortality^1^ as well as all-cause mortality in adulthood.^2^ Surprisingly little is known about the relationship between childhood socio-economic circumstances and dementia, even though low education, vascular risk factors and cardiovascular diseases (CVDs) are well-recognized potentially modifiable risk factors for dementia^3–5^ that are also rooted in early-life socio-economic circumstances.

Childhood socio-economic circumstances have consistently been shown to be associated with cognitive function across the life course,^6^^,^^7^ but studies regarding the rate of cognitive decline or clinical dementia are mixed.^6^^,^^8–10^ An increased risk of dementia has been observed among people who reported a lower childhood socio-economic position,^11–15^ a larger household or sibship size,^12^^,^^16^^,^^17^ stress or trauma^18^^,^^19^ or loss of a parent^20^ in some studies whereas not in others.^15^^,^^20–22^ Prior evidence is almost solely based on study participants’ or their relatives’ reports on childhood circumstances, which might introduce serious reporting bias. Especially retrospective recall data of childhood disadvantage is inaccurate and overestimates associations with many adulthood outcomes compared with prospectively collected data.^23^ We know of only two small-scale studies on dementia that have been able to utilize prospectively recorded data on childhood socio-economic circumstances, with contradictory results regarding the contribution of father’s occupational social class on dementia risk.^12^^,^^20^

Previous studies have shown attenuated associations between childhood socio-economic circumstances and dementia in models adjusting for adulthood education,^11–13^ but the underlying mechanisms have not been explicitly addressed. Disadvantaged childhood circumstances might increase the risk of dementia through impacting the structural and functional brain development,^24^ in which case childhood would reflect a particular critical period. Additionally, early-life socio-economic circumstances might set children on different trajectories in terms of socio-economic attainment or cardiovascular health, which will across the adulthood years contribute to the accumulation of cognitive reserve^25^ and the development of cerebrovascular burden affecting the risk of dementia.^26^

In this study, we analysed the association between childhood socio-economic circumstances and the risk of dementia, and investigated the mediating role of potentially modifiable risk factors including adulthood socio-economic position and cardiovascular health. Our study makes three unique contributions to the literature. First, we took advantage of a large population-based cohort using prospectively collected census and population register data on childhood and adulthood characteristics without self-report or attrition biases. Second, we employed mediation analysis to formally test adulthood socio-economic position and cardiovascular health as underlying mechanisms. Third, we analysed the associations separately for early-onset dementia to assess whether the childhood determinants differ according to age at dementia onset.^27^^,^^28^

## Methods

### Sample

The study employed register data of a 10% household sample drawn from the 1950 Finnish population census that Statistics Finland has linked to subsequent population registers using unique personal identification numbers assigned to all permanent residents in Finland. Statistics Finland further linked the sample with individual-level records of employment, health and mortality, and pseudonymized the data for analyses. We used data of children living in families at the age of 0–15 years at the time of the 1950 census (cohorts born in 1935–1950) and who were alive and lived in the community at the age of 49–64 years at the end of 1999 (*n* = 95 381). Delayed entry was allowed for returning migrants who entered the population between 2000 and 2017 (*n* = 674). Cohort members were followed up between 1 January 2000 and 31 December 2018 until the date of dementia diagnosis, death, emigration or the end of 2018, whichever came first.

### Dementia

Dementia was identified from several data sources including hospital, medication and death registers that cover the whole population. Dates of specialized outpatient or inpatient hospital care episodes with a dementia diagnosis [International Classification of Diseases (ICD) 10th Revision codes F00–03, F05.1 and G30] were collected from the hospital discharge register and patient censuses obtained from the Finnish Institute for Health and Welfare. Dates of all state reimbursed purchases of anti-dementia medication (Anatomical Therapeutic Chemical code N06D) and entitlement for special state reimbursement of anti-dementia medication costs (Finnish disease code 307) were collected from the medication reimbursement register of the Social Insurance Institution of Finland. The date and cause of death were obtained from the Death Register of Statistics Finland using ICD-10 codes F01–03 and G30. Dementia incidence was defined as the first entry in any of the aforementioned registers.

### Childhood socio-economic circumstances

Childhood socio-economic characteristics were obtained from the 1950 Finnish population census^29^^,^^30^ when the cohort members were 0–15 years old. We included characteristics that reflect the socio-economic position of the family, family structure and housing conditions. The classification of the variables derived from the census questionnaire and were coded based on previously published studies.^31^^,^^32^ The highest education of parents was classified as more than primary school (generally >6 years of education; International Standard Classification of Education ISCED-2011^33^ codes 2–8), primary school (6 years, ISCED 1) and less than primary school (<6 years, ISCED 0). Occupational social class of the head of the household was based on information of occupational status classified according to the International Standard Classification of Occupations (ISCO)^34^ and the size of farmland, categorized as non-manual, manual (including agriculture and forestry work), large farmer (with ≥10 hectares of field), small farmer (with <10 hectares of field), employer or other self-employed and other. Home ownership distinguished home owners, renters and unknown ownership. Geographical region of residence was classified as southern, western and eastern or northern Finland. Family type was classified as two-parent, single-mother and single-father family. Household crowding expressed the mean number of persons per heated room in the household, classified as <2, 2 to <3, 3 to <4, ≥4 and unknown. The standard of equipment indicated whether electric light, running water, sewerage, central heating, hot water, gas line, bathroom or toilet was present in the household. The standard of equipment was categorized as modest if at least two of these were present, poor if only one was present, very poor if none of these was present and unknown if no information was available. We also created an additional socio-economic index summarizing the childhood socio-economic circumstances. The variables included in the index were parents’ education, occupational social class, home ownership, household crowding and the eight housing assets as separate variables (0 = no, 1 = yes). Principal components analysis was used to summarize the variables in one index, retaining the first principal component and divided to quintiles.

### Adulthood mediators

The mediators included adulthood socio-economic position and cardiovascular health. The analysis of socio-economic position and cardiovascular health as separate mediators is warranted since a recent study indicated that adulthood socio-economic position and cardiovascular health influence dementia risk through distinct mechanisms.^35^ Socio-economic position was indicated by the highest attained education classified as tertiary (generally ≥13 years of education, ISCED 5–8), secondary (10–12 years, ISCED 3–4) and basic education (9 years, ISCED 0–2); occupational social class was categorized as non-manual, manual, self-employed farmer, other self-employed and other or unknown based on Statistics Finland classification of socio-economic groups;^36^ and household disposable income (in quintiles) adjusted for the household structure using the Organisation for Economic Co-operation and Development (OECD) modified equivalence scale. These characteristics were measured at baseline, with the exception of occupational social class, the information of which was collected from 1990, 1995, 2000 or 2005 depending on the baseline year. Because low income could indicate non-employment due to health-related factors, we also included the main type of economic activity at the age of 50 years, which distinguished the employed, unemployed and other non-employed (outside the labour market). For two cohorts economic activity was measured at the age of 49 years because the data were not available for the year 1986 and the youngest cohort turned 50 years old only after the baseline of the dementia follow-up. Because people with a low socio-economic position are less likely to be married compared with those with a high socio-economic position, and marital status groups also differ in terms of cardiovascular health, a time-varying measure of marital status was included and categorized as married, divorced, widowed and never married. The schematic directed acyclic graph in Supplementary Figure S1 (available as Supplementary data at *IJE* online) shows the proposed causal structure of this study.

Cardiovascular health was measured in terms of particular vascular risk factors and CVD. Vascular risk factors included alcohol-related diseases and accidental poisoning by alcohol, diabetes, dyslipidaemia and hypertension. Individuals with ischaemic heart disease, cerebrovascular disease, heart failure, atrial fibrillation or peripheral arterial disease were classified as having a CVD (for exact coding, see Supplementary Table S1, available as Supplementary data at *IJE* online). Vascular risk factors and CVD were identified using the registers of inpatient hospital care and medication reimbursements covering years from 1987 to 2017 (see Supplementary Table S1, available as Supplementary data at *IJE* online for details). The status of vascular risk factors and CVD (0 = no, 1 = yes) was updated during the follow-up.

### Statistical analysis

Dementia incidence rates per 1000 person-years at risk were first estimated by age and by all exposure and mediating variables. To evaluate the associations between childhood socio-economic circumstances and dementia, we used the Karlson–Holm–Breen (KHB) method, which allows separation of the effect of mediation or confounding from the effect of changing residual variance when comparing across multiple nested nonlinear probability models.^37^^,^^38^ We first fitted a discrete time survival model using logistic regression to estimate odds ratios (ORs) and 95% confidence intervals (CIs) for dementia by each childhood characteristic, adjusting for age, gender, region of residence in 1950 and calendar year in Model 1. In Model 2, all childhood characteristics were mutually adjusted, with the exception of childhood socio-economic index, which was modelled separately and adjusted for family type in Model 2.

We next analysed the extent of mediation through adulthood socio-economic position and cardiovascular health for childhood characteristics for which the 95% CI did not include 1.00 in Model 1. This mediation analysis applied the reformulated KHB method, which allows the introduction of mediators sequentially.^37^ The reformulated KHB method first estimates the linear predictor from the nonlinear (logistic) model, adjusted for all mediating and confounding variables (the so-called full model), after which the linear predictor is used as the dependent variable in nested ordinary least square (OLS) regression models first excluding and then including the mediators. The coefficients thus express the difference in the linear predictor between categories of exposure variables. Whereas the total effect model only included the childhood characteristic, the direct effect model simultaneously adjusted for all adulthood characteristics, and thus represents effect that is not mediated through adulthood socio-economic position or cardiovascular health. CIs for the mediation models were obtained using bootstrap with 1000 resamples of households. The proportion mediated was calculated as the indirect effect divided by the total effect. The same analyses were conducted for early-onset dementia only, censoring individuals at the end of the year they turned 65 years old.

We conducted a robustness analysis using Cox proportional hazards models, the results being highly similar (results available from the authors upon request). We tested for gender interaction between each childhood characteristic and dementia adjusting for covariates of Model 1, and since no interactions emerged (range of *P*-values 0.080–0.958), we analysed all models for men and women combined. All analyses were performed using Stata version 16.1.^39^

## Results

The characteristics of the study population and crude dementia incidence rates are summarized in Table 1. The mean age of the study population at baseline was 55.3 years (standard deviation 4.5) and 50% were women. Of the 95 381 individuals included in the analysis, 7059 individuals (7.4%) developed dementia between 2000 and 2018. The overall dementia incidence rate was 4.4 (95% CI 4.3–4.5) per 1000 person-years, the rate being substantially lower for dementia onset before the age of 65 years (0.9; 95% CI 0.8–1.0). The rate increased to 5.8 (95% CI 5.6–6.0) among those aged 65 to 74 years and to 23.6 (95% CI 22.8–24.6) at the age of ≥75 years (rates for these age groups not shown in the table). The incidence rate diverged between childhood socio-economic groups, being particularly high for cohort members with less than primary educated parents, unknown parental social class and those living with a single mother or a single father. A lower adulthood socio-economic position, non-employment, not being married and the presence of vascular risk factors or CVD were also related to a higher dementia incidence.

**Table 1:** Characteristics of the study population and incidence rate per 1000 person-years for overall dementia and early-onset dementia, Finnish men and women in 2000–2018

			Incidence rate (95% CI)			
	*n*	%	Overall dementia		Early-onset dementia	
**Age at baseline [mean (SD)]**	55.3 (4.5)					
**Gender**						
Men	47 861	50	4.4	(4.2–4.5)	0.9	(0.9–1.0)
Women	47 520	50	4.4	(4.3–4.6)	0.8	(0.8–0.9)
**Childhood circumstances**						
**Parents’ education**						
More than primary	9260	10	3.6	(3.3–3.9)	0.8	(0.7–1.0)
Primary	69 951	73	4.1	(4.0–4.3)	0.9	(0.8–0.9)
Less than primary	16 170	17	6.0	(5.8–6.3)	1.2	(1.0–1.4)
**Social class**						
Non-manual	14 185	15	3.9	(3.7–4.2)	0.8	(0.7–1.0)
Manual	41 848	44	4.0	(3.9–4.2)	0.9	(0.8–1.0)
Large farmer	7656	8	4.7	(4.4–5.1)	0.9	(0.7–1.2)
Small farmer	22 666	24	5.2	(4.9–5.4)	1.0	(0.8–1.1)
Employer/other self-employed	7409	8	4.3	(4.0–4.7)	0.8	(0.6–1.0)
Other/unknown	1617	2	5.8	(5.0–6.8)	1.3	(0.8–2.1)
**Home ownership**						
Own	58 580	61	4.7	(4.6–4.8)	0.9	(0.8–1.0)
Rent	30 171	32	3.8	(3.6–4.0)	0.8	(0.7–0.9)
Unknown	6630	7	4.5	(4.1–4.9)	1.0	(0.8–1.3)
**Region of residence**						
South	12 356	13	3.9	(3.6–4.1)	0.8	(0.6–1.0)
West	43 118	45	4.3	(4.1–4.4)	0.9	(0.8–1.0)
East and north	39 907	42	4.7	(4.5–4.9)	1.0	(0.9–1.1)
**Family type**						
Two-parent family	86 737	91	4.2	(4.1–4.3)	0.9	(0.8–1.0)
Mother only	7590	8	6.2	(5.8–6.7)	0.9	(0.6–1.1)
Father only	1054	1	8.3	(7.0–9.8)	1.5	(0.8–2.8)
**Household crowding** ^a^						
<2	30 952	32	4.2	(4.0–4.4)	0.8	(0.7–0.9)
2 to <3	30 396	32	4.2	(4.1–4.4)	0.9	(0.8–1.0)
3 to <4	15 687	16	4.7	(4.4–4.9)	0.8	(0.7–1.0)
≥4	17 271	18	4.9	(4.6–5.2)	1.1	(1.0–1.3)
Unknown	1075	1	3.4	(2.7–4.4)	1.6	(1.0–2.5)
**Standard of equipment**						
Modest	23 014	24	4.0	(3.9–4.3)	0.8	(0.7–0.9)
Poor	43 718	46	4.4	(4.3–4.6)	0.9	(0.8–1.0)
Very poor	27 702	29	4.7	(4.5–4.9)	1.0	(0.9–1.1)
Unknown	947	1	3.3	(2.5–4.3)	1.6	(1.0–2.6)
**Socio-economic index, quintile**						
Highest	18 996	20	4.0	(3.8–4.3)	0.8	(0.7–0.9)
4th	18 310	19	3.8	(3.6–4.1)	0.8	(0.7–1.0)
3rd	19 160	20	4.2	(4.0–4.5)	0.8	(0.7–0.9)
2nd	18 946	20	4.7	(4.5–5.0)	1.0	(0.8–1.1)
Lowest	19 969	21	5.2	(4.9–5.4)	1.1	(1.0–1.3)
**Adulthood socio-economic position**						
**Education**						
Tertiary	23 013	24	3.5	(3.3–3.6)	0.6	(0.6–0.8)
Secondary	29 272	31	3.8	(3.6–3.9)	0.8	(0.7–1.0)
Basic	43 096	45	5.4	(5.2–5.6)	1.1	(1.0–1.2)
**Occupational social class**						
Non-manual	43 295	45	3.8	(3.7–4.0)	0.6	(0.5–0.7)
Manual	34 146	36	5.0	(4.8–5.1)	1.0	(0.9–1.1)
Self-employed farmer	6945	7	4.8	(4.5–5.3)	0.6	(0.4–0.8)
Other self-employed	8106	8	3.9	(3.6–4.3)	0.9	(0.7–1.1)
Other/unknown	2889	3	8.3	(7.2–9.5)	8.4	(7.0–10.1)
**Household income, quintile**						
Highest	19 076	20	3.4	(3.2–3.6)	0.7	(0.6–0.8)
4th	19 076	20	3.4	(3.2–3.6)	0.7	(0.6–0.8)
3rd	19 075	20	4.2	(4.0–4.4)	0.8	(0.7–0.9)
2nd	19 076	20	5.3	(5.1–5.6)	1.0	(0.8–1.2)
Lowest	19 078	20	6.0	(5.7–6.3)	1.4	(1.3–1.6)
**Economic activity at age 50** **years**						
Employed	75 955	80	4.3	(4.2–4.4)	0.7	(0.7–0.8)
Unemployed	7098	7	3.6	(3.3–4.0)	1.3	(1.0–1.5)
Other non-employed	12 328	13	5.7	(5.4–6.1)	1.8	(1.6–2.1)
**Adulthood marital status** ^b^						
Married	63 820	67	3.8	(3.6–3.9)	0.7	(0.7–0.8)
Divorced	15 893	17	4.4	(4.2–4.7)	1.1	(1.0–1.3)
Widowed	4512	5	8.6	(8.1–9.1)	1.4	(1.1–1.8)
Never married	11 156	12	4.7	(4.3–5.0)	1.1	(0.9–1.3)
**Adulthood cardiovascular health** ^b^						
Alcohol-related conditions	2722	3	10.0	(9.2–10.9)	3.9	(3.3–4.7)
Diabetes	5424	6	8.3	(7.9–8.7)	1.5	(1.2–1.7)
Dyslipidaemia	10 463	11	7.2	(7.0–7.5)	1.2	(1.0–1.3)
Hypertension	37 465	39	5.9	(5.8–6.1)	1.1	(1.0–1.2)
CVD	10 151	11	9.1	(8.8–9.4)	1.8	(1.5–2.0)
**Total**	95 381	100	4.4	(4.3–4.5)	0.9	(0.8–1.0)

CVD, cardiovascular disease.

a Number of persons per heated room in household.

b Distribution according to status at baseline for time-dependent variables.

Adjusted for age, gender, region of residence in 1950 and calendar year (Table 2; Model 1), the risk of dementia was higher for cohort members who resided in eastern and northern parts of Finland compared with southern Finland (OR = 1.19, 95% CI 1.10–1.28), lived with a single father (OR = 1.27, 95% CI 1.07–1.51) or in a more crowded household (OR = 1.10, 95% CI 1.02–1.18 for 3 to <4 persons per heated room; OR = 1.19, 95% CI 1.11–1.27 for ≥4 persons). These associations were not attributable to other childhood characteristics, as adjusting mutually for all childhood characteristics in Model 2 had a negligible effect on the estimates. The ORs for dementia by adulthood socio-economic position and cardiovascular health are shown in Supplementary Table S2 (available as Supplementary data at *IJE* online).

**Table 2:** Associations between childhood socio-economic circumstances and dementia, Finnish men and women in 2000–2018

	Model 1: each variable separately^c^		Model 2: all childhood variables	
	OR	(95% CI)	OR	(95% CI)
**Parents’ education**				
More than primary	1.00		1.00	
Primary	1.00	(0.91–1.09)	0.96	(0.87–1.07)
Less than primary	1.07	(0.97–1.18)	1.01	(0.90–1.14)
**Social class of head of household**				
Non-manual	1.00		1.00	
Manual	1.04	(0.97–1.12)	1.01	(0.93–1.11)
Large farmer	0.90	(0.82–1.00)	0.92	(0.82–1.03)
Small farmer	1.03	(0.95–1.11)	1.02	(0.93–1.13)
Employer/self-employed	0.98	(0.88–1.10)	0.98	(0.87–1.10)
Other/unknown	1.17	(0.99–1.40)	1.12	(0.93–1.34)
**Home ownership**				
Own	1.00		1.00	
Rent	1.03	(0.98–1.09)	1.00	(0.94–1.07)
Unknown	1.07	(0.97–1.17)	1.06	(0.96–1.17)
**Region of residence**				
South	1.00		1.00	
West	1.06	(0.98–1.15)	1.08	(0.99–1.17)
East and north	1.19	(1.10–1.28)	1.18	(1.09–1.29)
**Family type**				
Two-parent family	1.00		1.00	
Mother only	1.07	(0.99–1.15)	1.05	(0.97–1.14)
Father only	1.27	(1.07–1.51)	1.26	(1.06–1.50)
**Household crowding^a^**				
<2	1.00		1.00	
2 to <3	1.03	(0.97–1.10)	1.03	(0.97–1.10)
3 to <4	1.10	(1.02–1.18)	1.10	(1.02–1.18)
≥4	1.19	(1.11–1.27)	1.18	(1.10–1.28)
Unknown	1.05	(0.81–1.36)	0.92	(0.49–1.72)
**Standard of equipment**				
Modest	1.00		1.00	
Poor	1.03	(0.97–1.10)	1.01	(0.94–1.08)
Very poor	1.01	(0.94–1.08)	0.94	(0.87–1.03)
Unknown	1.03	(0.77–1.36)	1.07	(0.54–2.12)
**Socio-economic index^b^**				
Highest	1.00		1.00	
4th	0.96	(0.89–1.04)	0.96	(0.88–1.04)
3rd	0.99	(0.92–1.07)	0.99	(0.92–1.07)
2nd	1.03	(0.95–1.11)	1.03	(0.95–1.11)
Lowest	1.03	(0.95–1.12)	1.03	(0.95–1.11)

OR, odds ratio.

a Number of persons per heated room in household.

b Socio-economic index adjusted for family type in Model 2.

c Model 1 adjusted for age, gender, region of residence in 1950 and calendar year.

The total effects of childhood socio-economic circumstances were next partitioned into indirect effects through adulthood socio-economic position and cardiovascular health, and into the direct effect not mediated by these factors (Table 3). The largest indirect effect was observed for the association between household crowding (3 to <4 and ≥4 persons per heated room) and dementia mediated through adulthood socio-economic position (47–65%). By contrast, most of the excess risk associated with single-father family and eastern and northern region of residence were direct, adulthood characteristics in concert mediating less than a third of the total effect at most. Socio-economic position mediated 16% and 17% of the associations of single-father family and region of residence with dementia, respectively. Smaller indirect effects mediated through cardiovascular health were observed for household crowding (10–15%) and eastern and northern region of residence (11%).

**Table 3:** Total, direct and indirect effects of childhood socio-economic circumstances through adulthood socio-economic position and cardiovascular health on dementia, and the proportion of total effect mediated (%), Finnish men and women in 2000–2018

	Total effect^b^			Indirect effect through adulthood socio-economic position^c^			Indirect effect through adulthood cardiovascular health^d^			Direct effect^e^	
	OR	Coef.	(95% CI)	Coef.	(95% CI)	%	Coef.	(95% CI)	%	Coef.	(95% CI)
**Region of residence**											
South	1.00	Ref.		Ref.			Ref.			Ref.	
West	1.06	0.06	(–0.02, 0.14)	0.01	(0.00, 0.02)		–0.01	(–0.01, 0.00)		0.06	(–0.02, 0.14)
East and north	1.19	0.17	(0.09, 0.25)	0.03	(0.02, 0.04)	17	0.02	(0.01, 0.02)	11	0.12	(0.04, 0.21)
**Family type**											
Two-parent family	1.00	Ref.		Ref.			Ref.			Ref.	
Mother only	1.07	0.07	(–0.01, 0.15)	0.02	(0.02, 0.03)		0.01	(0.00, 0.02)		0.04	(–0.04, 0.12)
Father only	1.27	0.21	(0.02, 0.40)	0.03	(0.02, 0.05)	16	–0.01	(–0.02, 0.01)	–2	0.18	(–0.01, 0.37)
**Household crowding^a^**											
<2	1.00	Ref.		Ref.			Ref.			Ref.	
2 to <3	1.03	0.03	(–0.03, 0.09)	0.03	(0.02, 0.05)		0.01	(0.00, 0.01)		–0.01	(–0.07, 0.05)
3 to <4	1.10	0.08	(0.01, 0.16)	0.05	(0.04, 0.07)	65	0.01	(0.01, 0.02)	15	0.02	(–0.06, 0.09)
≥4	1.19	0.16	(0.08, 0.23)	0.07	(0.05, 0.09)	47	0.02	(0.01, 0.02)	10	0.07	(–0.01, 0.14)
Unknown	1.05	0.02	(–0.26, 0.30)	0.05	(0.03, 0.07)		0.02	(0.00, 0.04)		–0.05	(–0.33, 0.23)

Coef., coefficient; OR, odds ratio.

a Number of persons per heated room in household.

b Odds ratios from logistic regression Model 1 in Table 2, regression coefficients express the difference in linear predictor obtained from the OLS model.

c Adulthood socio-economic position includes educational level, occupational social class, household income, economic activity at age 50 years and marital status.

d Adulthood cardiovascular health includes alcohol-related conditions, diabetes, dyslipidaemia, hypertension and CVD, with control for confounding by adulthood socio-economic position.

e Full model adjusting for all adulthood characteristics.

The associations between childhood socio-economic circumstances and early-onset dementia (*n* = 771) were similar to those for dementia overall (Table 4; Model 1). Likely because of the small number of dementia cases, however, only the 95% CIs of high (OR = 1.35, 95% CI 1.11–1.66 for ≥4 persons per heated room) and unknown household crowding (OR = 2.12, 95% CI 1.31–3.45) and unknown standard of equipment (OR = 2.21, 95% CI 1.26–3.58) did not include 1.00 in Model 1. As for overall dementia, the largest indirect effect in early-onset dementia was observed for high household crowding mediated through adulthood socio-economic position (55%) (Table 5). Only small indirect effects of household crowding and standard of equipment appeared to act through cardiovascular health (1–5%). The ORs for early-onset dementia by adulthood characteristics are shown in Supplementary Table S3 (available as Supplementary data at *IJE* online).

**Table 4:** Associations between childhood socio-economic circumstances and early-onset dementia (incident dementia cases *n* = 771), Finnish men and women in 2000–2018

	Model 1: each variable separately^c^		Model 2: all childhood variables	
	OR	(95% CI)	OR	(95% CI)
**Parents’ education**				
More than primary	1.00		1.00	
Primary	0.98	(0.77–1.25)	0.90	(0.68–1.19)
Less than primary	1.18	(0.89–1.57)	1.04	(0.75–1.44)
**Social class of head of household**				
Non-manual	1.00		1.00	
Manual	1.08	(0.87–1.34)	1.01	(0.79–1.30)
Large farmer	1.08	(0.78–1.49)	1.06	(0.74–1.52)
Small farmer	1.08	(0.85–1.37)	1.00	(0.75–1.34)
Employer/self-employed	0.90	(0.64–1.26)	0.88	(0.61–1.26)
Other/unknown	1.49	(0.89–2.49)	1.50	(0.87–2.58)
**Home ownership**				
Own	1.00		1.00	
Rent	0.97	(0.83–1.13)	0.97	(0.80–1.17)
Unknown	1.15	(0.88–1.50)	1.02	(0.75–1.37)
**Region of residence**				
South	1.00		1.00	
West	1.10	(0.87–1.39)	1.08	(0.85–1.38)
East and north	1.25	(0.99–1.59)	1.17	(0.91–1.50)
**Family type**				
Two-parent family	1.00		1.00	
Mother only	0.84	(0.62–1.14)	0.80	(0.58–1.10)
Father only	1.39	(0.74–2.59)	1.34	(0.72–2.50)
**Household crowding^a^**				
<2	1.00		1.00	
2 to <3	1.08	(0.90–1.30)	1.07	(0.89–1.30)
3 to <4	1.01	(0.81–1.27)	1.00	(0.79–1.26)
≥4	1.35	(1.11–1.66)	1.32	(1.06–1.64)
Unknown	2.12	(1.31–3.45)	1.93	(0.57–6.51)
**Standard of equipment**				
Modest	1.00		1.00	
Poor	1.10	(0.92–1.33)	1.09	(0.89–1.33)
Very poor	1.16	(0.94–1.43)	1.07	(0.84–1.37)
Unknown	2.12	(1.26–3.58)	1.14	(0.31–4.17)
**Socio-economic index^b^**				
Highest	1.00		1.00	
4th	1.01	(0.80–1.28)	1.01	(0.80–1.28)
3rd	0.97	(0.77–1.23)	0.97	(0.77–1.24)
2nd	1.14	(0.91–1.44)	1.15	(0.91–1.44)
Lowest	1.26	(1.00–1.59)	1.26	(1.00–1.58)

OR, odds ratio.

a Number of persons per heated room in household.

b Socio-economic index adjusted for family type in Model 2.

c Model 1 adjusted for age, gender, region of residence in 1950 and calendar year.

**Table 5:** Total, direct and indirect effects of childhood socio-economic circumstances through adulthood socio-economic position and cardiovascular health on early-onset dementia, and the proportion of total effect mediated (%), Finnish men and women in 2000–2018

	Total effect^b^			Indirect effect through adulthood socio-economic position^c^			Indirect effect through adulthood cardiovascular health^d^			Direct effect^e^	
	OR	Coef.	(95% CI)	Coef.	(95% CI)	%	Coef.	(95% CI)	%	Coef.	(95% CI)
**Household crowding^a^**											
<2	1.00	Ref.		Ref.			Ref.			Ref.	
2 to <3	1.08	0.08	(–0.09, 0.25)	0.08	(0.04, 0.11)		0.01	(0.00, 0.01)		0.00	(–0.18, 0.17)
3 to <4	1.01	0.00	(–0.23, 0.24)	0.11	(0.06, 0.16)		0.01	(0.00, 0.02)		–0.12	(–0.36, 0.12)
≥4	1.35	0.28	(0.07, 0.49)	0.15	(0.09, 0.21)	55	0.01	(0.01, 0.02)	5	0.11	(–0.10, 0.33)
Unknown	2.12	0.77	(0.25, 1.29)	0.10	(0.05, 0.15)	13	0.01	(–0.01, 0.03)	1	0.66	(0.14, 1.17)
**Standard of equipment**											
Modest	1.00	Ref.		Ref.			Ref.			Ref.	
Poor	1.10	0.11	(–0.09, 0.30)	0.08	(0.03, 0.12)		0.01	(0.00, 0.01)		0.03	(–0.17, 0.23)
Very poor	1.16	0.15	(–0.07, 0.36)	0.13	(0.06, 0.20)		0.01	(0.00, 0.01)		0.01	(–0.21, 0.24)
Unknown	2.12	0.77	(0.21, 1.34)	0.10	(0.05, 0.16)	13	0.01	(–0.01, 0.03)	2	0.66	(0.09, 1.22)

Coef., coefficient; OR, odds ratio.

a Number of persons per heated room in household.

b Odds ratios from logistic regression Model 1 in Table 4, regression coefficients express the difference in linear predictor obtained from the OLS model.

c Adulthood socio-economic position includes educational level, occupational social class, household income, economic activity at age 50 years and marital status.

d Adulthood cardiovascular health includes alcohol-related conditions, diabetes, dyslipidaemia, hypertension and CVD, with control for confounding by adulthood socio-economic position.

e Full model adjusting for all adulthood characteristics.

## Discussion

This study analysed the association between childhood socio-economic circumstances and later-life dementia, and assessed the mediating role of potentially modifiable risk factors including adulthood socio-economic position and cardiovascular health. The study took advantage of prospectively collected census and population register data, and thus avoided bias arising from inaccurate recollections of disadvantaged childhood circumstances. The results demonstrate that childhood disadvantage is a determinant of dementia; cohort members who lived in more crowded households, with a single father or in the eastern and northern parts of Finland as children faced an increased risk of dementia later in life. The findings thus add to the existing evidence on the ‘long arm of childhood’, previously shown for adulthood morbidity and mortality.^1^^,^^2^^,^^32^

To the best of our knowledge, this was the first study to employ formal mediation analysis to assess the magnitude of the indirect effects of childhood socio-economic circumstances on dementia risk through attained socio-economic position and cardiovascular health in adulthood, both of which are trajectories embarked upon already at early stages of life. The results show that adulthood socio-economic position in particular was a part of the mechanism linking household crowding in childhood to dementia risk. This finding is similar to previous investigations that have reported a higher risk of dementia associated with a greater number of siblings or larger household size^12^^,^^16^^,^^17^ and that these associations attenuated following adjustment for adulthood education.^12^^,^^17^ The lower socio-economic attainment of people with several siblings has been linked to resource dilution suggesting that a greater number of children in the family decreases the parental resources allocated to each child.^40^^,^^41^ To the extent that household crowding reflects the number of children in the family, the findings of this study indicate that fewer parental resources might lead to a higher risk of dementia acting through a lower socio-economic attainment.

The associations were not fully mediated by adulthood socio-economic position, however. In fact, the results indicate consistent direct effects of childhood socio-economic circumstances on dementia risk. This finding supports the ‘chain of risk’ life-course model and suggests that the risk accumulation begins already in early life. In particular, exposure to early-life disadvantage related to e.g. cramped households, poorer nutrition or stress might hamper brain development and cause long-lasting adverse consequences.^24^ Also in previous investigations, the associations between dementia and parental death in early childhood or adolescence,^20^ tooth loss before the age of 35 years potentially reflecting systemic inflammatory processes^11^ and shorter body growth reflecting childhood socio-economic or nutritional circumstances^11^^,^^15^^,^^42^^,^^43^ have been robust to adjustment for adulthood socio-economic position.

Our results also show a consistently higher risk of dementia for people who lived in eastern and northern Finland as children. Especially in the first half of the twentieth century, eastern and northern Finland were mostly sparsely populated rural areas and a higher prevalence of Alzheimer’s disease has been previously reported in these regions.^44^ Also in other countries a higher prevalence of Alzheimer’s disease has been linked with rural residence—an association that appears particularly clear in studies capturing rural residence in early life,^45^ although the underlying mechanisms are not clear. Our results indicate that the association was not attributable to the other socio-economic characteristics of the childhood family or to adulthood socio-economic position or cardiovascular health. Further research is warranted to identify the particular factors that bring about these regional differences.

A unique contribution of our study was that we were also able to study the social determinants of early-onset dementia. The results suggest that childhood socio-economic circumstances contribute similarly to early-onset dementia as to dementia overall. Although dementia incidence is much lower at younger ages compared with the later stages of life, early-onset dementia has substantial consequences on the lives of the affected and their families, including early exit from the labour market and premature death. Although it must be recognized that the causes of early-onset dementia are more varied than those of late-onset dementia,^28^ our results indicate that interventions to reduce childhood disadvantage might prevent or delay early exit from the labour market and postpone long-term care needs by reducing dementia incidence already at working age.

It is possible that the associations we observed in this study are specific to the cohorts who lived their childhood in the 1940s and 1950s—a period of high material deprivation and disadvantage in the wartime and post-war Finnish society. However, although younger cohorts have experienced substantial improvements in the standard of living and a decline in overall mortality, health inequalities in terms of childhood socio-economic circumstances seem not to have disappeared^46^ or have even strengthened for early- to mid-life mortality from substance-related and external causes of death.^47^ It thus appears plausible that despite the decline in the prevalence of risk-enhancing childhood factors for dementia, the relative inequalities may persist across cohorts. However, future studies to confirm cohort-specific associations between childhood socio-economic circumstances and dementia are needed.

Our register-based data have several advantages in studying older people and dementia in particular. Importantly, the data have been collected prospectively, covering the entire life course from childhood to older age. Thus our data are not subject to recall bias or inaccurate accounts of proxy reporters. Moreover, our data avoid bias arising from self-selection or selective attrition, which are common problems of prospective cohort studies of older people with cognitive impairment.^48^ Nevertheless, our study has some limitations. Population and health registers do not contain genetically informed data and thus we were unable to take into account genetic susceptibility to dementia. Although genetics may affect broad regional differentials in dementia, we have no clear evidence to believe that they might lie behind the association between housing conditions or family structure and dementia. Another limitation is that we could not observe health behaviours such as smoking, diet or physical activity directly. Although we could capture some of their effect indirectly through individuals’ medical histories, our measure of cardiovascular health may not fully capture all health-related risk factors across the life course. Finally, we could only identify people with dementia if their diagnosis was recorded in the hospital or death registers, or they had been prescribed anti-dementia medication. This is unlikely to have largely biased our results, as a validation study concluded that the aforementioned Finnish registers present good sensitivity and high precision for dementia diagnosis.^49^

## Conclusions

Our results support the view that childhood socio-economic circumstances are associated with later-life dementia risk—associations that are only partly attributable to potentially modifiable risk factors in adulthood including attained socio-economic position and cardiovascular health. The findings demonstrate that socio-economic and health interventions targeted at people of working age may not suffice in reducing the excess risk associated with disadvantaged childhood circumstances and thus intervention measures should be targeted at families with children.

## Ethics approval

The study was approved by Statistics Finland Board of Ethics and the Social and Health Data Permit Authority Findata (permit no. TK-53–1490-18 and THL/2180/14.02.00/2020). Participant consent was not required as administrative register data can be used for scientific purposes under the Personal Data Act and the Statistics Act. Statistics Finland pseudonymized the data prior to providing it to researchers.

## Supplementary Material

dyac205_Supplementary_Data

## Data Availability

The data that support the findings of this study are available from Statistics Finland, the Finnish Institute for Health and Welfare, and the Social Insurance Institution of Finland. Restrictions apply to the availability of these data, which were used under licence for this study. Data are available from the authors with the permission of Statistics Finland and the Social and Health Data Permit Authority Findata.
