# Supplementary material for: Childhood socio-economic circumstances and dementia: prospective register-based cohort study of adulthood socio-economic and cardiovascular health mediators
Source: Int J Epidemiol. 2022 Nov 7;52(2):523–35. doi: 10.1093/ije/dyac205 (PMC10114069; doi:10.1093/ije/dyac205)
Supplement: dyac205_Supplementary_Data [file dyac205_supplementary_data.pdf]

Supplementary material

Childhood socioeconomic circumstances and dementia: prospective register-based cohort study of adulthood socioeconomic and cardiovascular health mediators

Supplementary Figure S1 A schematic directed acyclic graph (DAG) showing the proposed causal structure of the study

Supplementary Table S1 International Classification of Diseases 9th and 10th Revision codes, Anatomical Therapeutic Chemical codes, and Finnish disease codes for vascular risk factors and cardiovascular disease

Supplementary Table S2 Associations between adulthood characteristics and dementia, Finnish men and women in 2000–2018

Supplementary Table S3 Associations between adulthood characteristics and early-onset dementia, Finnish men and women in 2000–2018

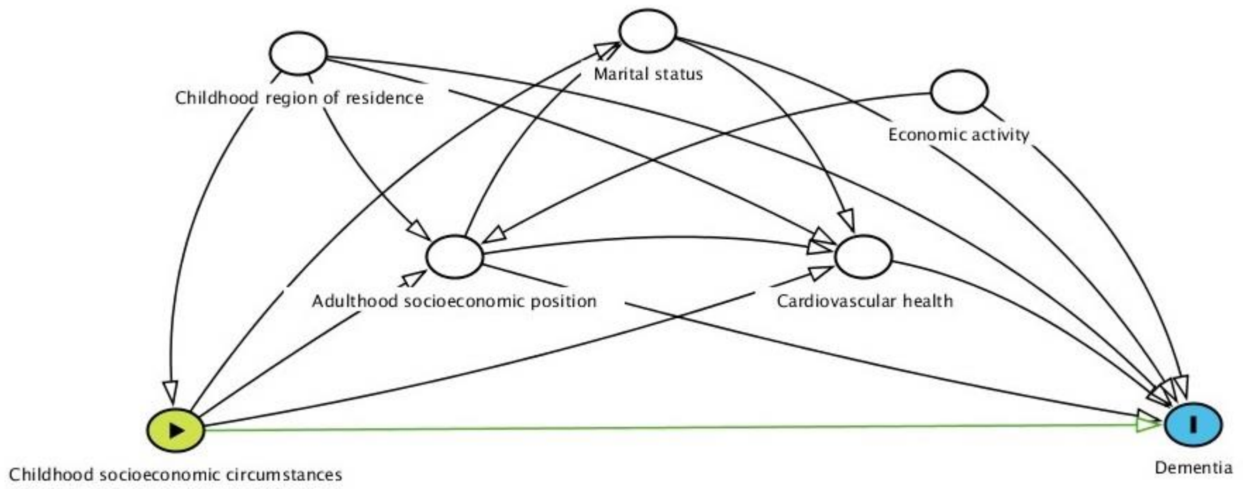

Supplementary Figure S1 A schematic directed acyclic graph (DAG) showing the proposed causal structure of the study. Adulthood socioeconomic position (education, occupational social class, and income) and cardiovascular health (alcohol-related diseases and accidental poisoning by alcohol, diabetes, dyslipidaemia, hypertension, and CVD) are regarded as en-bloc mediators. Childhood region of residence, marital status and economic activity are adjusted for as confounders (adjusted confounders gender, calendar year and age not shown for clarity). DAG created with dagitty.net.

Supplementary Table S1 International Classification of Diseases 9th and 10th Revision codes, Anatomical Therapeutic Chemical codes, and Finnish disease codes for vascular risk factors and cardiovascular disease

	ICD–9 (1987–1995)	ICD–10 (1996–2017)	ATC (1995–2017)	Finnish disease code (1987–2017)
Vascular risk factors				
Alcohol-attributable diseases and accidental poisoning by alcohol	291, 303, 3050, 3575, 4255, 5353, 5710–5713, 5770D–5770F, 5771C–5771D, E851	F10, G312, G4051, G612, G721, I426, K292, K70, K860, O354, X45		
Diabetes	250		E10–E14	A10
Dyslipidaemia	272	E78	C10	
Hypertension	401–405, 4160	I10–I13, I15, I270	C01–C05, C07–C09, B01	205
CVD				
Atrial fibrillation	4273	I48		
Cerebrovascular disease (including stroke)	430–438, 4378A	I60–I69, G45		
Heart failure	428	I50, I110, I130, I132		201
Ischaemic Heart Disease	410–414	I20–I25		
Peripheral arterial disease	440–448	I70–I77		206

ATC Anatomical Therapeutic Chemical; *CVD* cardiovascular disease; *ICD* International Classification of Diseases

Supplementary Table S2 Associations between adulthood characteristics and dementia, Finnish men and women in 2000–2018

	Model 1: each variable separately[a]		Model 2: all adulthood variables	
	OR	(95% CI)	OR	(95% CI)
Adulthood socioeconomic position				
Education				
Tertiary	1.00		1.00	
Secondary	1.12	(1.04–1.20)	1.02	(0.94–1.10)
Basic	1.20	(1.13–1.28)	1.06	(0.98–1.14)
Occupational social class				
Non-manual	1.00		1.00	
Manual	1.20	(1.13–1.26)	1.07	(1.01–1.14)
Self-employed farmer	0.95	(0.87–1.05)	0.88	(0.80–0.97)
Other self-employed	1.07	(0.97–1.17)	1.00	(0.91–1.11)
Other/unknown	2.37	(2.03–2.76)	1.91	(1.62–2.24)
Household income, quintile				
Highest	1.00		1.00	
4th	1.00	(0.92–1.08)	0.95	(0.88–1.04)
3rd	1.11	(1.02–1.20)	1.01	(0.93–1.10)
2nd	1.18	(1.09–1.27)	1.02	(0.94–1.11)
Lowest	1.38	(1.28–1.49)	1.10	(1.01–1.20)
Economic activity at age 50				
Employed	1.00		1.00	
Unemployed	1.41	(1.27–1.56)	1.14	(1.02–1.27)
Other non-employed	1.48	(1.38–1.58)	1.22	(1.13–1.31)
Adulthood marital status				
Married	1.00		1.00	
Divorced	1.26	(1.18–1.34)	1.13	(1.05–1.21)
Widowed	1.16	(1.09–1.25)	1.09	(1.02–1.17)
Never married	1.32	(1.22–1.43)	1.20	(1.11–1.30)
Adulthood cardiovascular health				
Alcohol-related conditions	2.95	(2.68–3.25)	2.41	(2.18–2.66)
Diabetes	1.45	(1.37–1.53)	1.22	(1.15–1.29)
Dyslipidaemia	1.34	(1.27–1.41)	1.13	(1.07–1.19)
Hypertension	1.45	(1.35–1.55)	1.24	(1.15–1.33)
CVD	1.61	(1.53–1.69)	1.35	(1.28–1.43)

CI confidence interval; *CVD* cardiovascular disease; *OR* odds ratio

[a] Adjusted for age, gender, region of residence in 1950, and calendar year

Supplementary Table S3 Associations between adulthood characteristics and early-onset dementia, Finnish men and women in 2000–2018

	Model 1: each variable separately[a]		Model 2: all adulthood variables	
	OR	(95% CI)	OR	(95% CI)
Adulthood socioeconomic position				
Education				
Tertiary	1.00		1.00	
Secondary	1.29	(1.05–1.58)	1.11	(0.89–1.39)
Basic	1.52	(1.25–1.84)	1.25	(1.00–1.55)
Occupational social class				
Non-manual	1.00		1.00	
Manual	1.51	(1.27–1.79)	1.27	(1.05–1.53)
Self-employed farmer	0.91	(0.62–1.32)	0.79	(0.54–1.17)
Other self-employed	1.41	(1.07–1.86)	1.26	(0.96–1.67)
Other/unknown	13.07	(10.47–16.31)	9.62	(7.31–12.65)
Household income, quintile				
Highest	1.00		1.00	
4th	1.01	(0.79–1.29)	0.92	(0.72–1.18)
3rd	1.12	(0.88–1.43)	0.93	(0.73–1.20)
2nd	1.26	(0.99–1.60)	0.97	(0.75–1.24)
Lowest	1.62	(1.28–2.04)	1.06	(0.81–1.38)
Economic activity at age 50				
Employed	1.00		1.00	
Unemployed	1.55	(1.22–1.96)	1.09	(0.85–1.40)
Other non-employed	1.96	(1.61–2.38)	1.38	(1.12–1.72)
Adulthood marital status				
Married	1.00		1.00	
Divorced	1.35	(1.12–1.61)	1.12	(0.92–1.35)
Widowed	1.53	(1.16–2.01)	1.35	(1.03–1.78)
Never married	1.24	(0.99–1.54)	1.01	(0.80–1.28)
Adulthood cardiovascular health				
Alcohol-related conditions	4.73	(3.83–5.83)	3.46	(2.78–4.31)
Diabetes	1.44	(1.18–1.77)	1.17	(0.94–1.44)
Dyslipidaemia	1.20	(1.02–1.40)	0.97	(0.81–1.16)
Hypertension	1.44	(1.23–1.68)	1.25	(1.05–1.48)
CVD	1.87	(1.59–2.20)	1.55	(1.28–1.86)

CI confidence interval; *CVD* cardiovascular disease; *OR* odds ratio

[a] Adjusted for age, gender, region of residence in 1950, and calendar year
